# Resveratrol Upregulates miR‐124‐3p Expression to Target DAPK1, Regulating the NLRP3/Caspase‐1/GSDMD Pathway to Inhibit Pyroptosis and Alleviate Spinal Cord Injury

**DOI:** 10.1111/jcmm.70338

**Published:** 2025-01-20

**Authors:** Daohui Li, Yongwen Dai, Zhengtao Li, Hangchuan Bi, Haotian Li, Yongquan Wang, Yuan Liu, Xinpeng Tian, Lingqiang Chen

**Affiliations:** ^1^ Department of Orthopedics The First Affiliated Hospital of Kunming Medical University Yunnan China; ^2^ Department of Critical Care Medicine Xi Chang People's Hospital Sichuan China

**Keywords:** microglia, miRNA‐124‐3p, pyroptosis, resveratrol, spinal cord injury

## Abstract

Currently, an effective treatment for spinal cord injury (SCI) is not available. Due to the irreversible primary injury associated with SCI, the prevention and treatment of secondary injury are very important. In the secondary injury stage, pyroptosis exacerbates the deterioration of the spinal cord injury, and inhibiting pyroptosis is beneficial for recovery from SCI. The aim of this study was to clarify the role of resveratrol (RES) and the antipyroptotic mechanisms of RES and miR‐124‐3p in SCI to lay a theoretical foundation for the clinical treatment of SCI and provide new therapeutic approaches. Using cell staining and related molecular protein detection techniques to assess DAPK1, the effects of miR‐124‐3p and RES on pyroptosis were investigated, and the effects of RES on injured spinal cord repair in rats were evaluated using tissue staining and related functional recovery experiments. In vitro, DAPK1 interacts with NLRP3, exerting a pyroptotic effect through the NLRP3/Caspase‐1/GSDMD pathway and DAPK1 knockdown inhibits pyroptosis. miR‐124‐3P negatively regulates the level of DAPK1 and reduced cell pyroptosis. RES increased miR‐124‐3p expression and reduces DAPK1 expression, affecting the NLRP3/Caspase‐1/GSDMD pathway and inhibiting pyroptosis. In vivo, RES reduces GSDMD‐N levels in rats with SCI, promotes functional recovery, and thus promotes recovery from SCI. Therefore, we concluded that RES increases the level of miR‐124‐3p, which targets DAPK1, regulates the NLRP3/Caspase‐1/GSDMD pathway, inhibits pyroptosis and alleviates SCI.

## 1 Introduction

Spinal cord injury (SCI) is a disease that severely affects human physical and mental health, quality of life and life expectancy. Surgery early after SCI can effectively improve nervous system function [1]. The subacute inflammatory response after SCI is mediated by increase in the levels of cytokines and chemokines secreted by resident microglia and astrocytes in the nervous system [2]. Microglia are common immune cells in the central nervous system and the earliest cells to react after SCI; they move toward lesions to play a protective role [3]. However, as the injury continues, stimulated microglia can undergo pyroptosis driven by TNF‐α. The leakage of proinflammatory factors such as IL‐1β can result in the secretion of a large amount of harmful substances, an increase in the occurrence of inflammatory reactions and thereby the exacerbation of SCI [4]. The production of cytokines and chemokines can lead to the extensive infiltration of immune cells, including microglia and neutrophils, and continue to produce additional inflammatory mediators similar to positive feedback effects [5]. Pyroptosis is a novel form of programmed cell death and has more specifically been defined as gasdermin protein family‐mediated programmed cell death. Gasdermin D (GSDMD) is a member of the gasdermin family that is active in forming pores in the cell membrane and a key effector molecule that mediates inflammatory cell death; it is known as the ‘cell pyroptosis executor’. The inflammatory response exacerbated by pyroptosis can lead to secondary spinal cord tissue degeneration and trigger a cascade of secondary injuries, further exacerbating SCI, which is the main cause of secondary SCI [6, 7, 8] (Figure S1). Therefore, pyroptosis plays an important role in the inflammatory response and is crucial for the treatment of SCI.

NOD‐like receptor family pyrin domain‐containing protein 3 (NLRP3) is a protein that plays a crucial role in the immune system, functioning as a key player in cellular signal transduction and inflammatory responses [9]. The NLRP3 inflammasome is a critical molecule for recognising injury in SCI [8], activating the inflammatory cascade, promoting the release of IL‐1β and IL‐18, and exacerbating spinal cord injury [10]. It also triggers pyroptosis, leading to cell membrane rupture and the release of inflammatory factors, further deteriorating inflammation and tissue damage [11]. NLRP3 not only directly affects neuronal survival and axonal regeneration but also acts on glial cells, creating a vicious cycle of inflammation and cell death [12, 13]. Given its significant role in inflammation and cell death, NLRP3 has become a key therapeutic target in the treatment of SCI.

Death‐associated protein kinase 1 (DAPK1) is a serine/threonine protein kinase regulated by calmodulin that is associated with apoptosis, autophagy, tumour growth and metastasis [14]; it is involved in regulating neuronal cell death in the nervous system [15] and is associated with various neurological diseases, such as cerebral ischaemia [16], stroke [17] and Alzheimer's disease [18]. In recent years, DAPK1 has been shown to be associated with inflammation and to have different anti‐inflammatory and proinflammatory effects on different research models [19, 20]. In addition, DAPK1 can bind to NLRP3 and is essential for the assembly of the NLRP3 inflammasome. The formation of the NLRP3 inflammasome is necessary for the production of active Caspase‐1 [21]. Active Caspase‐1 can cleave GSDMD to form N‐terminal fragments of GSDMD, which then create pores in the cell membrane, leading to pyroptosis [22]. According to previous studies, DAPK1 may exert antipyroptotic effects by influencing NLRP3/Caspase‐1/GSDMD; however, no research on the relationship between DAPK1 and pyroptosis has been reported.

MicroRNAs (miRNAs) are noncoding single‐stranded small‐molecule RNAs with a length of approximately 19–23 nucleotides [23]. Studies have shown that miRNAs are highly expressed in the central nervous system, including the brain and spinal cord [24]. Many studies have also shown that miRNAs are involved in inflammation, neuronal apoptosis, nerve regeneration and pyroptosis after SCI [25, 26, 27]. In a study by Mishima [28], RT‐PCR revealed that the concentration of miR‐124 in the central nervous system (cerebral cortex, cerebellum and spinal cord) of mice was more than 100 times higher than that in other organs, indicating the enrichment and specificity of miR‐124 in the central nervous system. A study by Miao [29] revealed that miR‐124‐3p affects pyroptosis by targeting CAPN1. As mentioned earlier, DAPK1 may be a molecular target that influences the pyroptosis, and related reports in the literature suggest that miR‐124‐3p targets DAPK1 [30]; and that miR‐124 may exert antipyroptotic effects by regulating DAPK1 expression and thus promotes injured spinal cord repair.

Resveratrol (RES), with the chemical formula C_14_H_12_O_3_, is a natural plant polyphenol antitoxin that has antioxidant, anti‐inflammatory and anticancer properties and protects cardiovascular health [31]. In recent years, studies have shown that RES has an antipyroptotic effect. For example, Tufekci [32] reported that RES can reduce pyroptosis in microglia through the Sirt1/AMPK pathway. Other studies indicate that RES can modulate the expression of miRNAs. For example, a study by Gandy [33] showed that RES can affect the miR‐124‐3p/SphK1 axis to ameliorate multiple sclerosis. Overall, RES has an antipyroptotic effect and can affect the expression of miR‐124‐3p. As mentioned earlier, DAPK1 may alter the regulation of pyroptosis via the NLRP3/Caspase‐1/GSDMD pathway, and miR‐124‐3p may target DAPK1. Therefore, RES may upregulate miR‐124‐3p expression and target DAPK1 to regulate the NLRP3/Caspase‐1/GSDMD pyroptosis pathway. However, the mechanism by which RES regulates pyroptosis in SCI has not yet been reported. The aims of this study were to reveal the mechanism by which RES inhibits pyroptosis in SCI, lay a theoretical foundation for the clinical treatment of SCI and provide new therapeutic options.

## 2 Materials and Methods

### 2.1 Cell Culture and Treatment

The mouse microglial cell line (BV2) was purchased from Saibaikang (Shanghai) Biotechnology Co. Ltd. (model: iCell‐m011, LOT:20230305). The cell culture medium was composed of high‐glucose DMEM (Gibco, USA), 10% fetal bovine serum (TransGen, China), 100 U/mL penicillin and 100 μg/mL streptomycin (Solarbio, China), and the cells were cultured at 37°C with 5% CO_2_ in an incubator. The cells were incubated with RES (Solarbio, China; 40 μM, 1 h) [32], followed by incubation with LPS (MedChemExpress, USA; 1 μg/mL, 5.5 h) and then ATP (MedChemExpress, USA; 5 mM, 0.5 h) [34] to verify the function of RES.

### 2.2 Animal Modelling and Treatment

Female Sprague–Dawley rats aged 8–12 weeks and weighing 180–220 g were used. An arterial clamp with a clamping force of 30 g was applied for 60 s to establish a stable SCI animal model. The rats with SCI were divided into an SCI + PBS group and an SCI + RES group, with three rats in each group. The concentration of RES was 20 mg/mL, and 20 mg of RES was directly mixed in 1 mL of PBS to prepare a turbid solution. Each rat was injected intraperitoneally (200 mg/kg/day) for 14 consecutive days [35]. Similarly, in the SCI + PBS group, an equal amount of PBS was injected intraperitoneally as a control. The animal experiments were conducted under conditions approved by the Animal Ethics Committee of Kunming Medical University and were conducted at the Animal Experiment Department of Kunming Medical University. In an SPF animal room (Kunming, China), standard feed and care were provided (ethics approval number: kmmu20230555).

### 2.3 Cell Transfection

A Transfection Kit (RiboBio, China) was used to transfect the siRNAs and miRNAs (RiboBio, China). The cells were transfected with 50 nM siRNA or miRNA in 24‐well plates. The cells were transfected at a confluence of 30%–40%. Thirty microliters of 1X riboFET TMCP buffer and 1.25 μL of 20 μM siRNA (or miRNA) storage solution were mixed gently, after which 3 μL of riboFET TMCP reagent was added. The mixture was shaken gently and incubated at room temperature for 15 min to prepare the transfection complex. Then, the complex was added to the cells in a complete culture medium supplemented with an appropriate amount of antibiotics and mixed well. After 24 h, transfection assessments and subsequent experiments were performed.

### 2.4 CCK8 Method for Measuring Cell Viability

The cells (1 × 10^5^) were seeded into a 24‐well plate to achieve the predetermined transfection (drug administration) conditions. At the end of the experiments, an appropriate amount of CCK8 reagent (Mei5bio, China) was added to each well, and the cells were incubated in a dark environment for 1–2 h. After the incubation, the reagent was gently aspirated and transferred to a 96‐well plate for subsequent analysis. The absorbance (OD value) of the cells was measured at 450 nm.

### 2.5 Extraction of Cell and Tissue Proteins

A mixture containing protease inhibitors (1:100), phosphatase inhibitors (1:100) and RIPA lysis buffer (EpiZyme, China) was prepared. Lysis buffer was added to the tissue or cells, which were subsequently ground until complete lysis occurred. The lysate was collected, and a cell ultrasonic crusher was used to mix and crush the cells. The samples were centrifuged at 12,000 rpm for 15 min in a high‐speed centrifuge to precipitate the cell debris and other solid substances. The supernatant was collected, and a BCA protein quantification kit (EpiZyme, China) was used to quantify the protein concentration. Loading buffer (EpiZyme, China) and enzyme‐free water were added to the samples, which were heated to 95°C–100°C for 10–15 min to denature the protein. The samples were stored at −80°C for future experimental use.

### 2.6 Western Blot

Protein samples were separated via sodium dodecyl sulphate–polyacrylamide gel electrophoresis (SDS–PAGE) (EpiZyme, China) at 250 V for 35 min. The separated proteins were transferred to 0.45 μm polyvinylidene fluoride membranes (Millipore, USA) in rapid transfer solution (EpiZyme, China) at 400 mA for 15–30 min; for macromolecular proteins, the membrane transfer time was increased appropriately. The membranes were blocked in a protein‐free rapid blocking solution (EpiZyme, China). After 5 min of blocking, the membranes were incubated with primary antibodies against DAPK1 (ABclonal, 1:1000), NLRP3 (Beyotime, 1:1000), Cleaved caspase‐1 (Affinity, 1:1000), GSDMD‐N (ABclonal, 1:500) and β‐actin (EpiZyme, 1:10,000) at room temperature on a shaker for 2–4 h. The membranes were then incubated with the corresponding secondary antibody (Biodragon, 1:10,000) for 1–2 h. An ultrasensitive chemiluminescence assay kit (EpiZyme, China) and Bio‐Rad development machine were used, and the protein band intensity was analysed using Image Lab and ImageJ software.

### 2.7 RT‐qPCR

A Total RNA Extraction Kit (TransGen, China) was used for total RNA extraction and miRNA purification strictly according to the manufacturer's instructions. A reverse transcription kit (TransGen, China) was subsequently used to synthesise cDNA. RT‐qPCR analysis was performed with Taq Pro Universal SYBR qPCR Master Mix (Vazyme, China); the expression levels were determined based on the threshold cycle (*C*_t_), and relative expression levels were calculated using the $2^{-\Delta \Delta C_{t}}$ method. U6 served as an internal reference.

### 2.8 Dual‐Luciferase Gene Reporter Assay

The target cells were transfected with X‐Tremegane HP transfection reagent (Roche, Switzerland). The transfection reagent and plasmid were dissolved in 100 μL of Opti‐MEM (Gibco, USA), and the mixture was added evenly to the cells, after which the cells were incubated. Fluorescence labelling was assessed under a fluorescence microscope to determine whether the transfection was successful. After 48 h, a Dual‐Luciferase Reporter Assay System (Promega, USA) was used for to analyse the cells. An appropriate amount of passive lysis buffer was used to lyse the cells. First, Luciferase Assay Reagent was added to the wells of Maxisorp plates, and then; the sample was added, followed by mixing. A microplate reader was used to measure the fluorescence value of firefly luciferase within 5 min. Stop&Glo reagent was added the mixture was allowed to react at room temperature for 3 min, and a microplate reader was used to measure the fluorescence value of Renilla luciferase. An analysis of the ratio of firefly/Renilla fluorescence values was performed to obtain the relative expression level of luciferase.

### 2.9 Hoechst 33342/YO‐PRO‐1 Staining

The cells were collected in a 1.5 mL centrifuge tube, the supernatant was removed, and an appropriate amount of cell staining buffer (Solarbio, China) was added to resuspend the cell pellet. Five microliters of Hoechst staining solution (Solarbio, China) was added, and 5 μL of YO‐PRO‐1 staining solution (Beyotime, China) was added. The cells and staining solutions were gently mixed and then incubated in an ice bath at 4°C for 20–30 min. The cells were subsequently pelleted, washed once with PBS, placed on slides and observed with a fluorescence microscope.

### 2.10 ELISAs

ELISAs were performed with ELISA kits (Beyotime, China). The supernatants of the cell cultures were added to precoated wells in a plate. The reaction wells were covered with a film (transparent), followed by an incubation at room temperature for 2 h. After five washes, biotinylated antibodies were added to the wells, and the reaction wells were covered with a film (transparent), followed by an incubation at room temperature for 1 h. After five washes, streptavidin‐labelled horseradish peroxidase was added to the wells. The reaction wells were covered with a film (white), followed by an incubation in the dark at room temperature for 20 min. After 5 washes, the chromogenic agent TMB (solution) was added to the wells. The reaction wells were covered with a film (white), followed by an incubation in the dark at room temperature for 20 min. Termination solution was added to the wells, followed by mixing. The A450 value was measured immediately after mixing. The corresponding concentration of each sample was calculated based on its absorbance value and a standard curve.

### 2.11 Immunoprecipitation

The source of the reagent kit used was Beyotime, China. The culture supernatant was collected. The antibody preparation solution was prepared. The antibody was combined with Protein A + G agarose beads, followed by gentle rotation and then an incubation. The sample was washed three times. Finally, the Protein A + G agarose beads were resuspended in TBS. The Protein A + G agarose beads bound to antibodies or normal IgG were washed with SDS–PAGE buffer and centrifuged, and the supernatant was subjected to SDS–PAGE and Western blotting.

### 2.12 HE, Nissl and Immunofluorescence Staining of Spinal Cord Tissue

#### 2.12.1 HE Staining

Fresh spinal cord tissue samples were placed in a fixative solution and fixed for 24 h. After being washed overnight with tap water, the tissue were dehydrated using a standard dehydration procedure and embedded in paraffin. The paraffin‐embedded tissue were sectioned (5 μm thick) and stained. The sections were dewaxed with xylene I and xylene II for 15 min each; then, the sections were washed with anhydrous ethanol and 95% anhydrous ethanol for 5 min each. Next, gradient dehydration was performed with 90% ethanol, 80% ethanol and 70% ethanol for 3 min each. Finally, the sections were stained with distilled water and a haematoxylin dye solution for 3 min and 2 s, respectively. Next, the sections were incubated with an eosin dye solution for 5 min and then washed with anhydrous ethanol and xylene for 5 min each. The sections were sealed with neutral resin. The staining results were observed under a microscope, and images were captured.

#### 2.12.2 Nissl Staining

Paraffin sections were routinely dewaxed with water as described earlier. The sections were subsequently immersed in Nissl staining solution (Beyotime, China) at 50°C–60°C for 20–40 min. The sections were washed with distilled water. Rapid differentiation was subsequently performed with 95% ethanol. Further dehydration was carried out with anhydrous ethanol, and then the sections were cleared using xylene and sealed with neutral resin. The stained sections were observed under a microscope.

#### 2.12.3 Immunofluorescence Staining of Spinal Cord Tissue

The paraffin sections were dewaxed and hydrated, followed by three washes with PBS buffer. The citric acid method was used for antigen retrieval in a 100°C oven for 45 min. After natural cooling, the sections were washed three times with PBS again. The sections on the glass slide were outlined using a chemical pen. The sections were incubated with 1% Triton‐100 for 10 min to permeabilise the cell membranes, after which the sections were shaken dry and incubated with H_2_O_2_ for 15 min to block endogenous peroxidases. The sections were subsequently washed with water. Afterward, the sections were incubated with a primary antibody against GSDMD‐N (ABclonal, 1:50) overnight at 4°C. When the sections were at room temperature, they were washed three times with PBS and then incubated with a secondary antibody (ProteinTech, 1:100) at room temperature in the dark for 1 h. The sections were then washed three times with PBS and stained with DAPI (Solarbio, China) for 2 min, followed by three washes with PBS. The Neutral resin was used to seal the sections before they were observed under a fluorescence microscope.

### 2.13 Basso‐Beattie‐Bresnahan (BBB) Scores

BBB behavioural scoring was performed preoperatively and on days 1, 3, 7 and 14 postoperatively. The BBB scores is used to evaluate the motor function of rats, and the highest possible score is 21 points. The higher the score is, the better the motor ability of the rats. Conversely, the worse the score is, the more severe the nerve damage. During the scoring process, the rats were placed on a specific platform, and their performance was recorded using various indicators.

### 2.14 Footprint Analysis

For the footprint analysis of rats in the SCI + PBS group and SCI + RES group, the forelimbs were stained with blue paint, and the hindlimbs were stained with red paint. The rats were placed on white paper with barriers along the sides, allowing the rats to walk in a straight line and facilitating the assessment of the recovery of hind limb function.

### 2.15 Statistical Analysis

The data were analysed using GraphPad Prism 9.5.1 software. Before the intergroup difference were analysed, normality distribution and homogeneity of variance tests were performed on the data. Differences between groups were analysed via one‐way ANOVA and paired *t* tests. When *p* < 0.05, the difference was considered statistically significant. All experiments were independently repeated at least three times.

## 3 Results

### 3.1 Establishment of a Pyroptosis Model and Assessment of the Expression of Related Molecules

**FIGURE 1:**
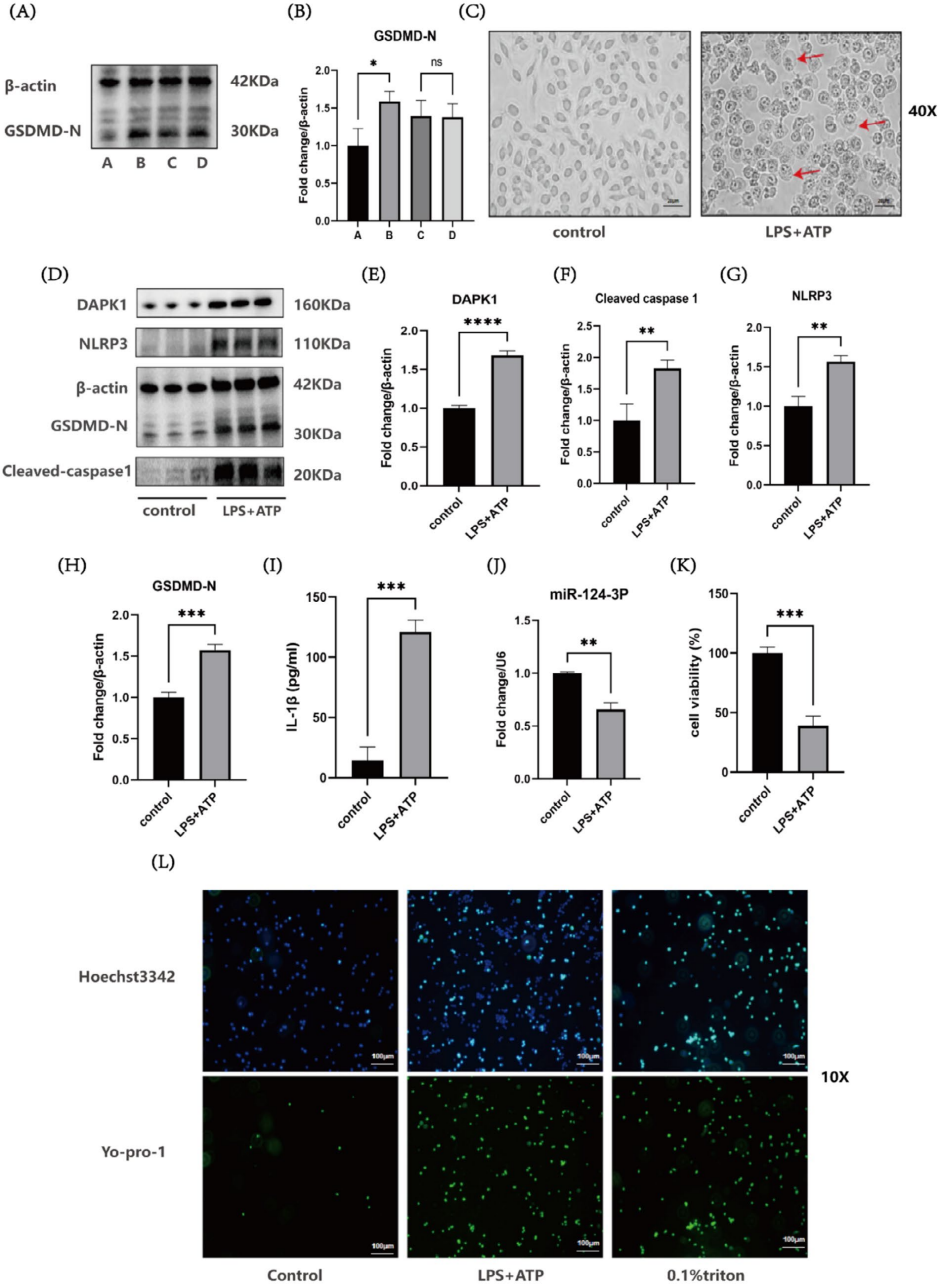
Establishment of a cellular pyroptosis model to assess the level of miR‐124‐3p, DAPK1, NLRP3, Cleaved caspase‐1, and GSDMD‐N. (A, B) Different pyroptosis induction schemes and GSDMD‐N protein levels. (C) Images of changes in cell morphology; captured with an inverted microscope; scale bar: 20 μm. (D–H) Western blot analysis of protein levels in the control and LPS + ATP groups. (I) ELISA of the IL‐1β concentration in the control and LPS + ATP groups. (J) RT‐qPCR analysis of miR‐124‐3p levels in the control and LPS + ATP groups. (K) A CCK‐8 assay was used to assess cell viability in the control and LPS + ATP groups. (L) Hoechst 3342/Yo‐pro‐1 staining was performed to observe the degree of pyroptosis in the control, LPS + ATP, and positive control groups; scale bar: 100 μm. (*p < 0.05, **p < 0.01, ***p < 0.001, ****p < 0.0001 and ns: not significant).

LPS + ATP was used to construct a pyroptosis model. The following times and concentrations were used: Group A‐control group; Group B‐LPS (1 μg/mL, 5.5 h) + ATP (5 mM, 0.5 h); Group C‐LPS (1 μg/mL, 2 h) + ATP (5 mM, 2 h); and Group D‐LPS (1 μg/mL, 1 h) + ATP (5 mM, 0.5 h). The level of the pyroptosis‐related protein GSDMD‐N was assessed by Western blot, with Group B exhibiting the highest level (*p* < 0.05). This concentration and duration were selected for subsequent experiments with the pyroptosis model (Figure 1A,B). After modelling, significant changes in cell morphology were observed under an inverted microscope, with the appearance of a membrane‐like structure (indicated by the red arrow), further confirming the successful construction of the pyroptosis model (Figure 1C). Compared with those in the control group, the protein expression levels of DAPK1 (*p* < 0.0001), NLRP3 (*p* < 0.01) and GSDMD‐N (*p* < 0.001) were significantly higher in the LPS + ATP group. The levels of pyroptosis‐related proteins, such as Cleaved caspase‐1 (*p* < 0.01), indicated significant pyroptosis (Figure 1D–H). Compared with the control group, the LPS + ATP group presented a higher concentration of the inflammatory factor IL‐1β (*p* < 0.001), further supporting the successful establishment of the pyroptosis model and indicating that pyroptosis leads to an increase in the concentration of the inflammatory factor IL‐1β (Figure 1I). RT‐qPCR was used to measure the changes in miR‐124‐3p expression after modelling, and compared with that in the control group, the expression level of miR‐124‐3p was significantly lower in the LPS + ATP group (*p* < 0.01), indicating a decrease in the miR‐124 level after pyroptosis was induced (Figure 1J). The CCK‐8 assay results revealed that cell viability was significantly lower in the LPS + ATP group than in the control group (*p* < 0.001), indicating a decrease in cell viability in the pyroptosis model (Figure 1K). By utilising the pore‐forming characteristics of pyroptosis and using Hoechst/Yo‐pro‐1 double staining, cells with disrupted membranes were directly observed, which indirectly reflect the degree of pyroptosis. The results revealed that the LPS + ATP group had more cells with disrupted membranes than did the control group and the number was roughly the same as the 0.1% Triton group (positive control group). These findings further indicate that pyroptosis was induced in the LPS + ATP group (Figure 1L).

### 3.2 Knocking Down DAPK1 Affects the NLRP3/Caspase‐1/GSDMD Pathway and Inhibits Pyroptosis

**FIGURE 2:**
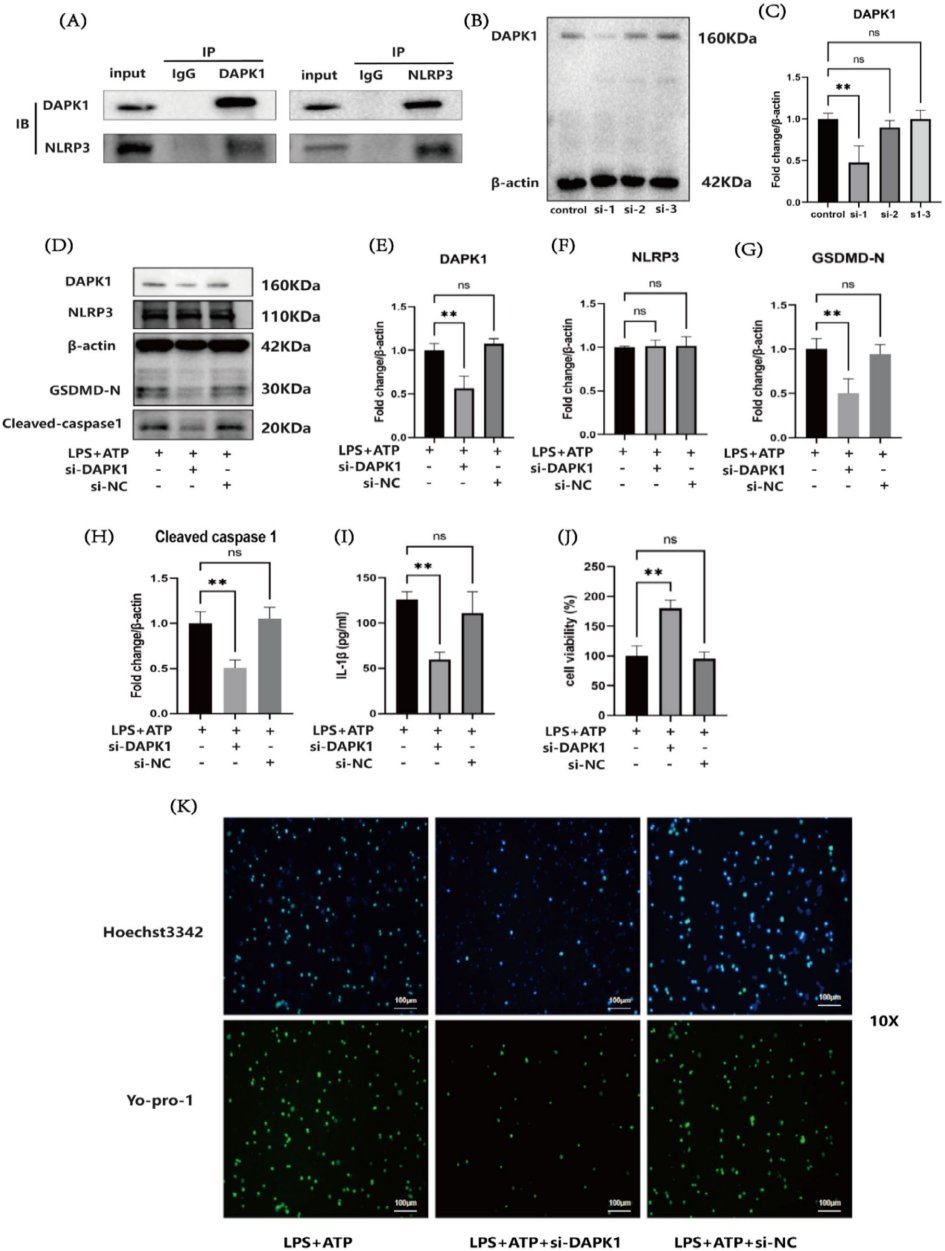
Interaction between DAPK1 and NLRP3 and the impact of DAPK1 knockdown on the protein levels of NLRP3, Cleaved caspase‐1 and GSDMD‐N. (A) Interaction between DAPK1 and NLRP3. (B, C) Western blot analysis of the effect of si‐DAPK1 knockdown. (D–H) Western blot analysis of protein levels in the LPS + ATP, LPS + ATP + si‐DAPK1 and LPS + ATP + si‐NC groups. (I) ELISA of the IL‐1β concentration in the LPS + ATP, LPS + ATP + si‐DAPK1 and LPS + ATP + si‐NC groups. (J) A CCK8 assay was performed to assess cell viability in the LPS + ATP, LPS + ATP + si‐DAPK1 and LPS + ATP + si‐NC groups. (K) Hoechst 3342/Yo‐pro‐1 staining was performed to observe the degree of pyroptosis in the LPS + ATP, LPS + ATP + si‐DAPK1 and LPS + ATP + si‐NC groups; scale bar: 100 μm (**p < 0.01, ns: not significant).

The immunoprecipitation results revealed the presence of the DAPK1 and NLRP3 proteins in the input group. The IgG group served as a negative control group, and no relevant proteins were detected via Western blot. Next, an anti‐DAPK1 antibody was used for precipitation and pull‐down. The Western blot results indicated the presence of DAPK1 and NLRP3, and reverse validation was performed using an anti‐NLRP3 antibody for precipitation and pull‐down. The Western blot results indicated the presence of DAPK1 and NLRP3, indicating that DAPK1 interacted with NLRP3 (Figure 2A). Owing to the upregulation of DAPK1 protein expression after pyroptosis, three siRNAs targeting DAPK1 were constructed and labelled as si‐1, si‐2 and si‐3 to investigate the effect of interfering with DAPK1 on pyroptosis. The three siRNAs were transfected into cells, and the knockdown effects were assessed via Western blot. The results revealed a significant difference (*p* < 0.01) between the si‐1 and control groups, indicating that the knockdown effect of si‐1 was good. Therefore, si‐1 was selected as the si‐DAPK1 for subsequent experiments (Figure 2B,C). Changes in the levels of pyroptosis‐related proteins after DAPK1 knockdown were assessed via Western blot analysis. Compared with the LPS + ATP group, the LPS + ATP + si‐DAPK1 group presented significantly lower levels of DAPK1 (*p* < 0.01), GSDMD‐N (*p* < 0.01) and Cleaved caspase‐1 (*p* < 0.01), but a significant difference in NLRP3 expression was not observed (*p* > 0.05), indicating that knocking down DAPK1 did not affect the transcription of NLRP3 but affected the formation of the NLRP3 inflammasome, resulting in a decrease in the level of Cleaved caspase‐1 protein and a decrease in the level of the pyroptosis executor protein GSDMD‐N. These findings suggest that knocking down DAPK1 can affect the NLRP3/Caspase‐1/GSDMD pathway to combat pyroptosis (Figure 2D–H). ELISA was used to determine the concentration of IL‐1β. Compared with the LPS + ATP group, the LPS + ATP + si‐DAPK1 group presented a significantly lower concentration of the inflammatory factor IL‐1β (*p* < 0.01), indicating that knocking down DAPK1 can reduce the pyroptosis‐induced increase in the concentration of the inflammatory factor IL‐1β (Figure 2I). A CCK8 assay was performed to assess cell viability, and the results revealed that, compared with the LPS + ATP group, the LPS + si‐DAPK1 group presented a greater number of viable cells (*p* < 0.01), indicating that knocking down DAPK1 can decrease pyroptosis and increase cell viability (Figure 2J). Hoechst 3342/YO‐PRO‐1 staining revealed fewer cells with disrupted membranes in the LPS + ATP + si‐DAPK1 group than in the LPS + ATP group. Knocking down DAPK1 can reduce the degree of pyroptosis (Figure 2K).

### 3.3 Effects of miR‐124 Targeting DAPK1 on Pyroptosis

**FIGURE 3:**
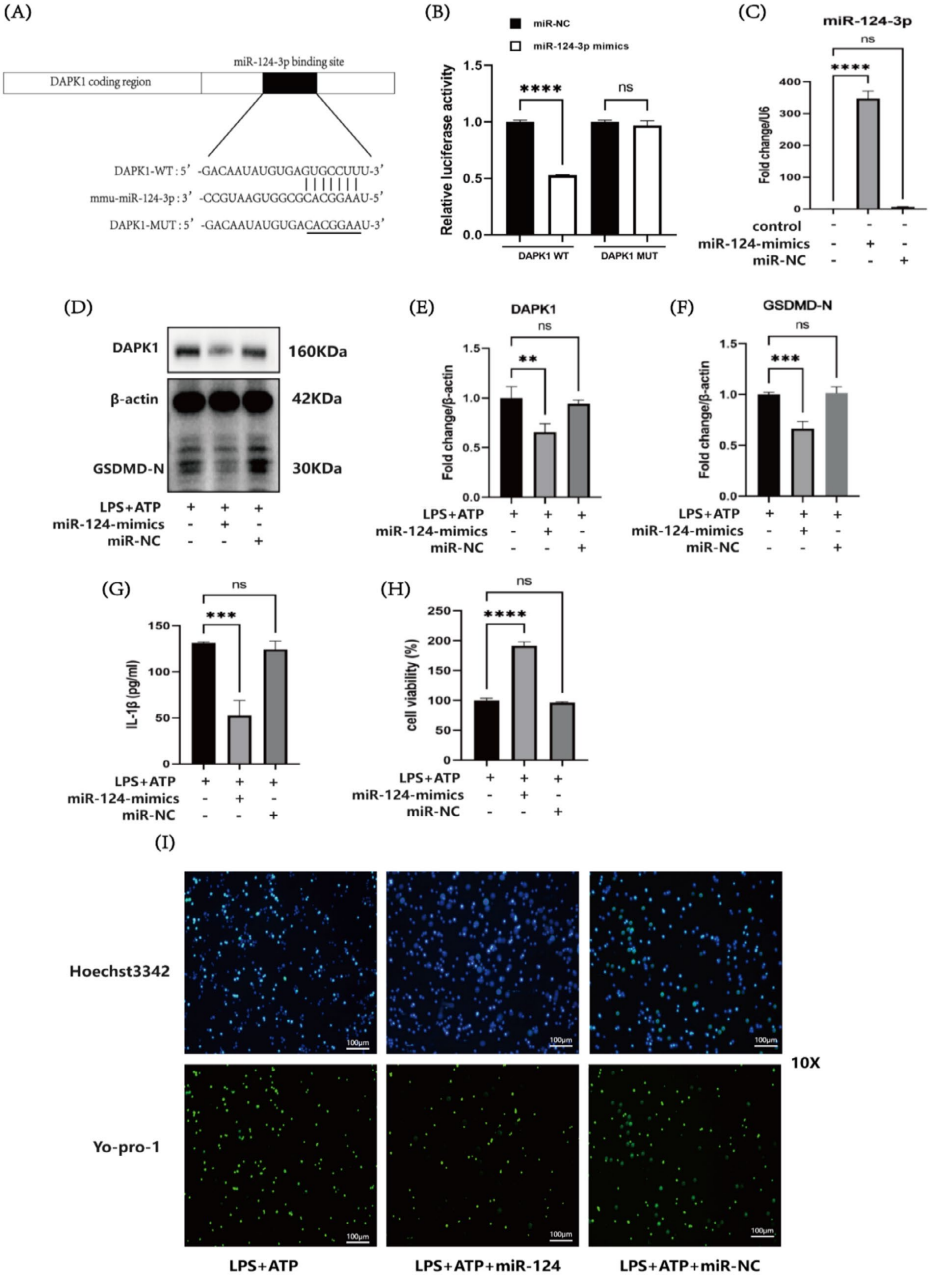
The interaction between miR‐124‐3p and DAPK1 alters GSDMD‐N levels to counteract pyroptosis. (A) Prediction of binding sites between DAPK1 and miR‐124‐3p from gene analysis websites. (B) Dual‐luciferase reporter assay to validate the targeting relationship between DAPK1 and miR‐124‐3p. (C) RT‐qPCR measurement of changes in miR‐124‐3p expression. (D–F) Western blot analysis of protein levels in the LPS + ATP, LPS + ATP + miR‐124‐mimic and LPS + ATP + miR‐NC groups. (G) ELISA of the IL‐1β concentration in the LPS + ATP, LPS + ATP + miR‐124‐mimics and LPS + ATP + miR‐NC groups. (H) A CCK8 assay was performed to assess cell viability in the LPS + ATP, LPS + ATP + miR‐124‐mimic and LPS + ATP + miR‐NC groups. (I) Hoechst 3342/Yo‐pro‐1 staining was performed to observe the degree of pyroptosis in the LPS + ATP, LPS + ATP + miR‐124‐mimic and LPS + miR‐NC groups; scale bar: 100 μm (**p < 0.01, ***p < 0.001, ****p < 0.0001 and ns: not significant).

The results from the miRNA and target gene prediction website ‘starbase.sysu.edu.cn’ indicated binding sites for miR‐124‐3p in the DAPK1 sequence (Figure 3A). a DAPK1 mutant was constructed (Figure 3A), and the interaction between DAPK1 and miR‐124‐3p was validated through dual‐luciferase experiments to verify the accuracy of the prediction. The results revealed that the WT + miR‐124‐3p mimic group significantly differed from the WT + miR‐NC group (*p* < 0.0001). A statistically significant difference was not observed (*p* > 0.05) between the MUT + miR‐124‐3p mimics group and the MUT + miR‐NC group. These findings indicate the presence of targeted binding sites between DAPK1 and miR‐124‐3p, which is consistent with the predicted results (Figure 3B). The transfection efficiency of miR‐124‐3p was determined by RT‐qPCR, and the results revealed that, compared with that in the control group, the expression level of miR‐124‐3p in the transfection group was significantly higher (*p* < 0.0001). A significant difference was not observed in the miR‐NC group (*p* > 0.05), indicating the successful transfection of miR‐124‐3p (Figure 3C). The effects of miR‐124‐3p transfection on the levels of the DAPK1 protein and the pyroptosis‐related protein GSDMD‐N were determined by Western blot analysis. Compared with those in the LPS + ATP group, the expression of DAPK1 (*p* < 0.01) and the pyroptosis executor protein GSDMD‐N (*p* < 0.001) was significantly lower in the LPS + ATP + miR‐124 mimics group. A dual‐luciferase assay revealed that miR‐124‐3p negatively regulated the expression of DAPK1. These findings also indicate that the upregulation of miR‐124‐3p expression has an antipyroptotic effect on cells (Figure 3D–F). ELISAs were performed to determine the concentration of IL‐1β. Compared with the LPS + ATP group, the LPS + ATP + miR‐124 mimic group exhibited a significantly lower concentration of the inflammatory factor IL‐1β (*p* < 0.01), indicating that miR‐124‐3p can reduce the pyroptosis‐induced increase in the concentration of the inflammatory factor IL‐1β (Figure 3G). A CCK8 assay was conducted to assess cell viability, and the results revealed that, compared with the LPS + ATP group, the viability of the LPS + ATP + miR‐124 mimic group was significantly increased (*p* < 0.0001), indicating that miR‐124‐3p can improve cell viability in the pyroptosis model (Figure 3H). Hoechst 3342/YO‐PRO‐1 staining revealed that the LPS + ATP + miR‐124 mimic group contained fewer cells with disrupted membranes. The transfection of miR‐124‐3p reduced the degree of pyroptosis (Figure 3I).

### 3.4 RES Upregulates miR‐124‐3p Expression and Targets DAPK1 to Regulate the NLRP3/Caspase‐1/GSDMD Pathway, Reducing Microglial Pyroptosis

**FIGURE 4:**
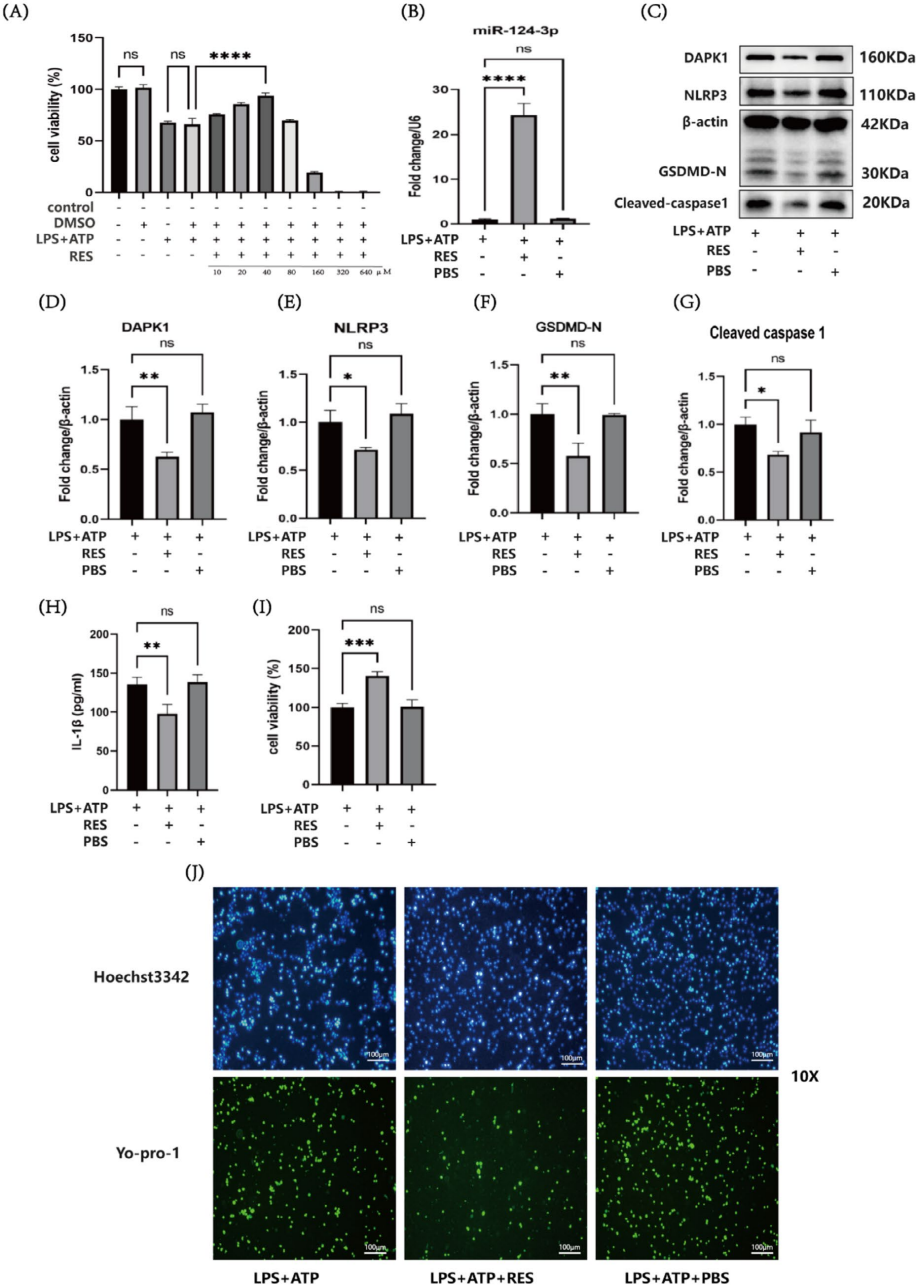
The upregulation of miR‐124‐3p expression by RES affects DAPK1, NLRP3, Cleaved caspase‐1 and GSDMD‐N protein levels to inhibit pyroptosis. (A) CCK8 assay showing peak cell viability after exposure to RES at a concentration of 40 μM. (B) RT‐qPCR analysis of changes in miR‐124‐3p levels caused by RES. (C–G) Western blot analysis of changes in protein levels in the LPS + ATP, LPS + ATP + RES and LPS + ATP + PBS groups. (H) ELISA of the IL‐1β concentration in the LPS + ATP, LPS + ATP + RES and LPS + ATP + PBS groups. (I) A CCK8 assay was performed to assess cell viability in the LPS + ATP, LPS + ATP + RES and LPS + ATP + PBS groups. (J) Hoechst 3342/Yo‐pro‐1 staining showing the degree of pyroptosis in the LPS + ATP, LPS + ATP + RES and LPS + ATP + PBS groups; scale bar: 100 μm (*p < 0.05, **p < 0.01, ***p < 0.001, ****p < 0.0001 and ns: not significant).

This experiment used different RES concentrations (10, 20, 40, 80, 160, 320 and 640 μM). The CCK8 assay results revealed that there was no significant difference (*p* > 0.05) between the control group and the DMSO group or between the LPS + ATP group and the LPS + ATP + DMSO group, indicating that the concentrations of DMSO used did not affect cellular activity and could be used as a solvent for RES. When the RES concentration was 40 μM, the peak cell viability was observed (*p* < 0.0001); therefore, subsequent experiments were conducted with RES at a concentration of 40 μM (Figure 4A). RT‐qPCR was used to determine the level of miR‐124‐3p. Compared with that in the LPS + ATP group, the expression level of miR‐124‐3p in the LPS + ATP + RES group was significantly higher (*p* < 0.0001), indicating that RES can upregulate the expression of miR‐124‐3p (Figure 4B). Compared with the LPS + ATP + RES group, the expression levels of DAPK1 (*p* < 0.01), NLRP3 (*p* < 0.05), Cleaved caspase‐1 (*p* < 0.05) and pyroptosis‐related proteins such as GSDMD‐N (*p* < 0.01) were lower in the other groups (Figure 4C–G). ELISAs were performed to measure the concentration of IL‐1β. Compared with the LPS + ATP group, the LPS + ATP + RES group presented a lower concentration of the inflammatory factor IL‐1β (*p* < 0.01), indicating that RES can reduce the pyroptosis‐induced increase in the IL‐1β concentration (Figure 4H). Compared with the LPS + ATP group, the LPS + ATP + RES group presented greater cell viability (*p* < 0.01), indicating that RES attenuated the decrease in cell viability caused by LPS + ATP‐induced pyroptosis (Figure 4I). Hoechst3342/YO‐PRO‐1 staining revealed that the degree of pyroptosis in the LPS + ATP + RES group was reduced (Figure 4J). Based on previous experiments, RES increased the level of miR‐124‐3p, and miR‐124‐3p negatively regulated DAPK1 expression. Reduced DAPK1 expression can affect the NLRP3/Caspase‐1/GSDMD pathway, thereby reducing the occurrence of pyroptosis. In other words, RES upregulates miR‐124‐3p expression and negatively regulates DAPK1 expression, affecting the NLRP3/Caspase‐1/GSDMD pathway and thereby reducing the occurrence of pyroptosis.

### 3.5 RES Improves Functional Recovery From SCI in Rats by Inhibiting Pyroptosis

**FIGURE 5:**
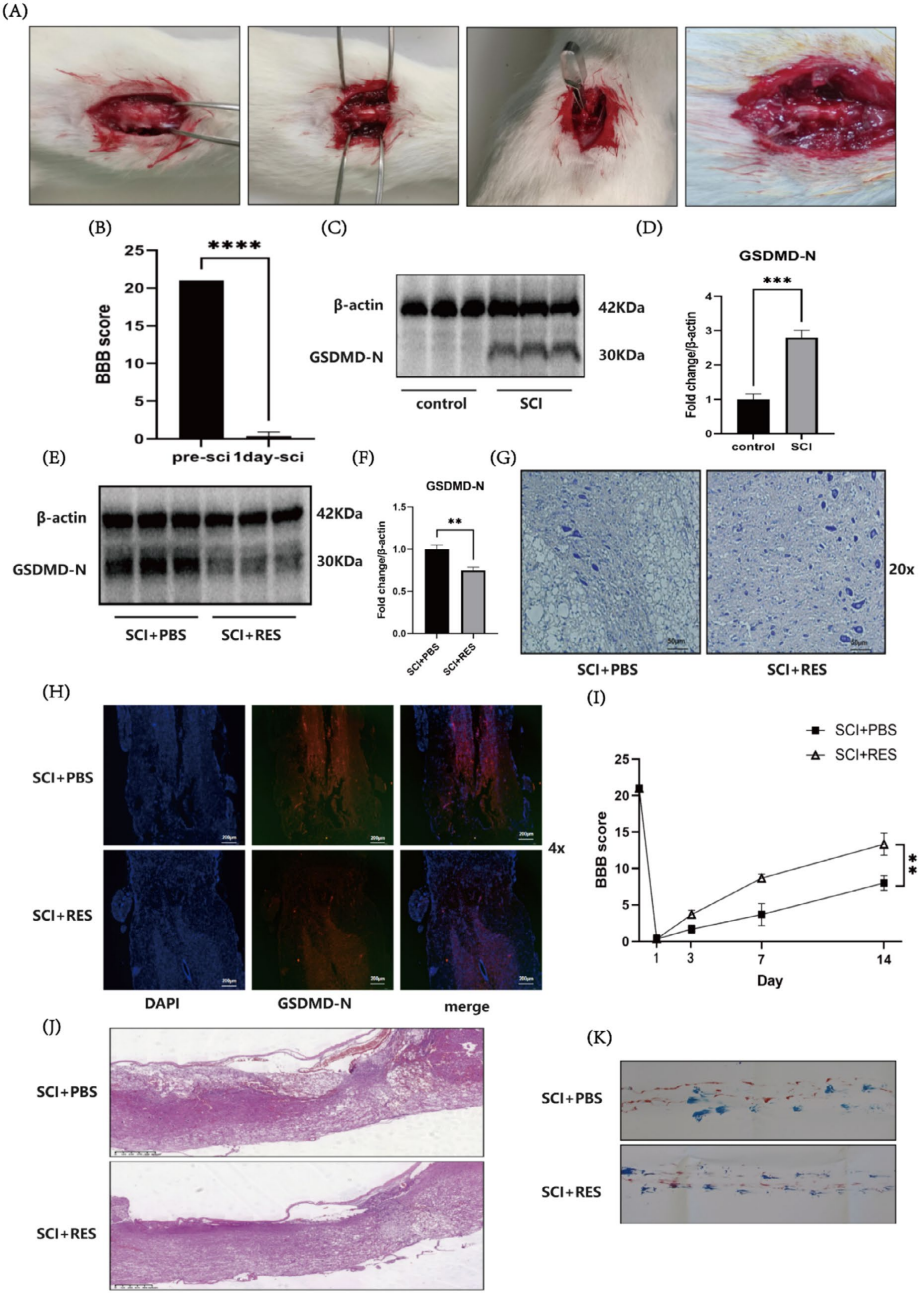
Establishment of a stable SCI model via compression, significant upregulation of GSDMD‐N levels in the SCI rat model, and the RES‐mediated reduction in GSDMD‐N levels and improvement in functional recovery in rats with SCI. (A) Process for establishing the SCI model via compression. (B) BBB scoring method to confirm the successful construction of the SCI animal model. (C, D) Western blot analysis of GSDMD‐N levels in the control and SCI groups. (E, F) Western blot analysis of GSDMD‐N protein levels in the SCI + RES and SCI + PBS groups. (G) Nissl staining showing the Nissl bodies in the SCI + RES and SCI + PBS groups; scale bar: 50 μm. (H) Immunofluorescence staining of spinal cord tissue showing the fluorescence intensity of GSDMD‐N in the SCI + PBS and SCI + RES groups; scale bar: 200 μm. (I) Comparison of BBB scores between the SCI + PBS and SCI + RES groups. (J) HE staining showing the spinal cord morphology in the SCI + RES and SCI + PBS groups; scale bar: 625 μm. (K) Footprint analysis of the SCI + PBS and SCI + RES groups (**p < 0.01, ***p < 0.001 and ****p < 0.0001).

A stable SCI animal model was established via the clamp method (clamping force of 30 *g* for a duration of 60 s) (Figure 5A). BBB behavioural scoring was performed to determine whether the SCI animal model was successfully established. Compared with those in the pre‐SCI group, the BBB behavioural scores of the rats in the 1‐day SCI group were significantly lower (*p* < 0.0001), indicating the successful establishment of the SCI animal model (Figure 5B) Western blot was used to assess protein expression at the SCI site in each group of rats, and the results revealed a significant increase in the level of the pyroptosis‐related protein GSDMD‐N in the SCI group (*p* < 0.001), indicating that pyroptosis occurred in the SCI group (Figure 5C,D). As a method to determine the effect of RES on SCI in rats, Western blot analysis was conducted to determine the changes in the level of GSDMD‐N, a pyroptosis executor protein, from rats in the SCI + RES group. Compared with that in the SCI + RES group, the level of GSDMD‐N in the SCI + RES group was significantly lower (*p* < 0.01), indicating that RES can reduce the occurrence of pyroptosis after SCI in rats (Figure 5E,F). Nissl staining revealed that the number of Nissl bodies in the SCI + RES group was significantly greater than that in the SCI + PBS group, indicating that RES can prevent the reduction in Nissl bodies in rats after SCI (Figure 5G). The immunofluorescence staining of spinal cord tissue revealed a lower fluorescence intensity of GSDMD‐N protein in the SCI + RES group than in the SCI + PBS group, indicating that RES can reduce the occurrence of pyroptosis in rats after SCI (Figure 5H). A significant difference in the BBB score was observed between the SCI + RES group and the SCI + PBS group on the 14th day (*p* < 0.01), indicating that RES promoted, the recovery of hind limb function in rats after SCI to some extent (Figure 5I). Haematoxylin/eosin staining revealed that the spinal cord and cells in the SCI + PBS group were severely damaged, with obvious clamp marks and numerous vacuoles. Compared with the SCI + PBS group, the SCI + RES group presented fewer vacuoles and a better recovery of the spinal cord morphology, indicating that RES promoted morphological recovery after SCI (Figure 5J). In the footprint experiment, blue dye was used to stain the forelimbs. The blue paw prints were spread out and evenly spaced, indicating that the forelimbs were not injured during the modelling process and, suggesting normal forelimb function. The red dye was used to stain the hind limbs and the hindlimbs (red) of the rats in the SCI + PBS group presented drag marks, indicating the poor recovery of hind limb function. The hindlimbs (red) of the rats in the SCI + RES group presented no obvious drag marks or intervals, indicating the good recovery of hind limb function and that RES promoted the recovery of hind limb function in the rats with SCI (Figure 5K).

## 4 Discussion

In recent years, many studies have shown that pyroptosis plays an important role in secondary SCI injury [36]. Pyroptosis is an inflammatory form of cell death that differs from the traditional forms of apoptosis and necrosis. During the process of pyroptosis, many proinflammatory factors and the cell contents are released, triggering a strong inflammatory response [37]. Following SCI, pyroptotic neurons and glial cells release inflammatory factors such as tumour necrosis factor and interleukins, exacerbating the inflammatory response and leading to secondary damage [38]. Therefore, inhibiting pyroptosis has become a potential method for treating SCI. According to reports, RES has anti‐inflammatory antioxidant [39] and antipyroptotic effects [40] and can affect miRNA expression, which in turn can influence the activation or inhibition of related pathways, indicating that it can regulate the expression of genes involved in the occurrence, development and prognosis of SCI [41]. Exploring the impact and mechanism of RES on SCI can contribute to the treatment of patients with SCI.

In the study of SCI, the BV2 cell line is often used as a model system to study the mechanism of secondary injury [42], and research has shown that BV2 cells can release inflammatory factors upon stimulation, causing an inflammatory response and leading to secondary damage [43]. Therefore, the BV2 cell line plays an important role in studying the mechanisms of SCI and seeking treatment options. Many studies have used BV2 cells as a model for pyroptosis in SCI, such as Kong [44] and Liu [45], who used LPS + ATP‐stimulated BV2 cells to simulate an SCI‐induced pyroptosis model. BV2 cells are commonly used to study central nervous system diseases; therefore, BV2 cells were selected as the experimental cells for studying pyroptosis in SCI in this study.

DAPK1 is a protein that plays an important role in regulating cell apoptosis and survival and has also been found to be closely associated with inflammation [46]. According to the literature, a close relationship exists between DAPK1 and central nervous system diseases [47]. For example, a study by Pei suggested that the loss of DAPK1 expression and the loss of the DAPK1 kinase domain can protect mice from neuronal and spinal cord injury, respectively, caused by stroke [48]. In addition, studies have shown that DAPK1 can regulate the activation of inflammasomes such as NLRP3, potentially affecting the occurrence of pyroptosis [49], There is a lack of research on the regulatory mechanism of DAPK1 in pyroptosis following SCI. Therefore, the aim of our study was to determine whether DAPK1 affects the NLRP3 inflammasome to regulate the occurrence of pyroptosis. First, we established a pyroptosis model in BV2 cells using LPS and ATP and found that the expression of DAPK1 increased, consistent with the increased expression of DAPK1 in the context of neuronal cell death mentioned above. It is interesting to note that the literature has reported that LPS activates NFkB through TLR4, which typically inhibits DAPK1 transcription [50]. Under the experimental conditions of LPS + ATP, not only did the expression of DAPK1 in cells not decrease, but it increased instead. Based on its known functions and related signalling pathways, we speculate that the possible reasons for the upregulation of DAPK1 in the LPS + ATP‐induced pyroptosis model are as follows: Firstly, there is a difference in cell types, such as LPS stimulation increasing the expression of DAPK1 and inflammatory markers in THP‐1 cells [51]; Secondly, there is a complex signalling regulatory network, where in addition to activating the NF‐κB pathway, LPS also activates other signalling pathways (such as the MAPK pathway) [52]. Activation of the MAPK (mitogen‐activated protein kinase) pathway can upregulate the expression of DAPK1; Lastly, it could be due to the feedback regulation of inflammatory factors and the role of ATP. There is literature indicating that the increase of inflammatory factors such as IL‐1β and TNF‐α can promote the expression of DAPK1 [19, 49]. ATP is not only an inducer of pyroptosis but can also, through its receptors (such as the P2X7 receptor), activate other signalling pathways (such as the MAPK pathway) [53, 54], indirectly affecting the expression of DAPK1. In summary, the upregulation of DAPK1 expression results from the combined effects of multiple signalling pathways and the cellular environment. Even though NF‐κB typically suppresses its transcription, other factors may still lead to increased DAPK1 expression under different conditions.

Moreover, we confirmed through immunoprecipitation experiments that DAPK1 can interact with NLRP3. Western blot analysis revealed that after DAPK1 was knocked down, the levels of Cleaved caspase‐1 and GSDMD‐N decreased in the cellular pyroptosis model. The decrease in the IL‐1β concentration detected by ELISA confirmed that DAPK1 interacts with NLRP3 and does not affect the expression level of NLRP3 but rather affects the activation of the NLRP3 inflammasome, which in turn inhibits pyroptosis through the NLRP3/Caspase‐1/GSDMD pathway.

miR‐124‐3p is a short noncoding RNA, and researchers widely believed that miR‐124‐3p is highly expressed in the central nervous system, including during the maturation and differentiation of neuronal cells. These findings have contributed to the treatment and diagnosis of neurological diseases [55]. For example, Jiang reported that the miR‐124‐3p/MYH9 axis can regulate and activate microglia through the PI3K/AKT/NF‐κB pathway, affecting SCI repair [56]. In addition, miR‐124‐3p is associated with pyroptosis, which can affect the stability of the lncRNA MALAT1 and inhibit chondrocyte pyroptosis, thereby reducing cartilage damage [57]. Overall, miR‐124‐3p is closely related to central nervous system diseases, including spinal cord injury and is associated with the occurrence of pyroptosis. In addition, miR‐124‐3p can interact with DAPK1, thereby regulating gene expression and apoptosis. For example, miR‐124 can target DAPK1 to inhibit neuronal cell death caused by ischemic stroke in mice [58], and miR‐124 can regulate the expression of DAPK1 and affect cell apoptosis in Parkinson's disease [59]. In this study, the expression of miR‐124‐3p and DAPK1 was assessed in SCI and pyroptosis models using RT‐qPCR and Western blot, respectively. The results revealed that the expression of miR‐124‐3p was significantly reduced, whereas the expression of DAPK1 was increased. Moreover, the dual‐luciferase results revealed a binding site for miR‐124‐3p in the DAPK1 sequence. We constructed miR‐124‐3p mimics and transfected them into cells to investigate the effect of miR‐124‐3p on the mechanism of SCI in vitro. Through CCK8 assays and Western blot analysis, we found that the upregulation of miR‐124‐3p expression increased the viability of SCI‐induced cells and inhibited pyroptosis. Western blot analysis revealed that the upregulation of miR‐124‐3p decreased DAPK1, NLRP3, Cleaved caspase‐1 and GSDMD‐N levels. The decrease in the concentration of the inflammatory factor IL‐1β detected by ELISA indicated that miR‐124‐3p negatively regulates the expression of DAPK1 and has an antipyroptotic effect on microglia. This study revealed that miR‐124‐3p has an antipyroptotic effect, which is consistent with the results of other studies. Combined with the previously mentioned reduction in DAPK1 expression, miR‐124‐3p can affect the NLRP3/Caspase‐1/GSDMD pathway to inhibit the occurrence of pyroptosis, indicating that miR‐124‐3p negatively regulates DAPK1 to exert antipyroptotic effects.

Recent studies have shown that RES, which is an efficient anti‐inflammatory substance, inhibits the inflammatory response of the spinal cord after injury by activating the SIRT‐1/NF‐κB signalling pathway [60]. Additionally, resveratrol can exert its effects by regulating ion channels, such as K^+^, Ca^2+^ and Na^+^ channels [61, 62, 63, 64]. Following SCI, ion channels also undergo changes, leading to increased intracellular sodium and calcium concentrations, as well as elevated extracellular potassium levels [65, 66, 67, 68]. These findings suggest that resveratrol may influence the repair of SCI by regulating ion channels. Moreover, studies have shown that RES has an antipyroptotic effect. For example, RES can reduce retinal ischaemia–reperfusion injury By effectively inhibiting the activity of the NLRP3/Gasdermin D/Caspase‐1/Interleukin‐1β pyroptosis pathway, RES plays an important role in protecting the retina from inflammatory responses [69]. The results of the aforementioned studies indicate that RES may play an important role in promoting injured spinal cord repair and inhibiting pyroptosis. Owing to the scarcity of research papers on the antipyroptotic effects of RES, the specific mechanism by which RES inhibits pyroptosis in SCI has not yet been reported. Therefore, the main purpose of this study was to elucidate the antipyroptotic mechanism of RES in SCI. Our team compared the effects of RES and PBS and then added RES to the LPS + ATP‐induced pyroptosis model to explore whether RES affects the NLRP3/CASP1/GSDMD pathway by increasing miR‐124‐3p expression and targeting DAPK1 to inhibit microglial pyroptosis and promote injured spinal cord repair. The RT‐qPCR analysis revealed that RES upregulates the expression of the miR‐124‐regulated protein DAPK1. The differential expression levels of NLRP3, Cleaved caspase‐1 and GSDMD‐N decreased, the concentration of the inflammatory factor IL‐1β, as detected by ELISA, significantly decreased, and Hoechst/YO‐PRO‐1 staining revealed a decrease in the degree of cell membrane rupture. Cell viability, as measured by the CCK‐8 assay, also increased, indicating that RES can affect the levels of miR‐124‐3p and pyroptosis pathway‐related proteins to inhibit pyroptosis. Combined with the findings of previous experiments indicating that miR‐124‐3p negatively regulates DAPK1 expression and; DAPK1 interacts with NLRP3, affecting the NLRP3/Caspase‐1/GSDMD pathway to inhibit pyroptosis, our results indicate that RES can upregulate miR‐124‐3p expression and negatively regulate the influence of DAPK1 on the NLRP3/ Caspase‐1/GSDMD pathway to exert antipyroptotic effects. We also conducted in vivo experiments. In a rat SCI model, Western blot and immunofluorescence analyses of spinal cord tissue revealed that RES can reduce the levels of GSDMD‐N, indicating that RES also has an antipyroptotic effect in vivo. Moreover, the results of the BBB score, footprint experiments, HE staining and Nissl staining indicated that RES promoted recovery from SCI.

In summary, the results of this study indicate that DAPK1, NLRP3, Cleaved caspase‐1 and GSDMD‐N were highly expressed in an in vitro SCI pyroptosis model, whereas miR‐124‐3p was expressed at low levels. Immunoprecipitation experiments revealed that DAPK1 can interact with NLRP3. After DAPK1 was knocked down, the levels of Cleaved caspase‐1 and GSDMD‐N decreased, but no significant change in the expression level of NLRP3 was observed, indicating that DAPK1 interacts with NLRP3 and alters the activation of the NLRP3 inflammasome, thereby modulating the NLRP3/ Caspase‐1/GSDMD pathway to inhibit pyroptosis. A dual‐luciferase assay confirmed the existence of binding sites for miR‐124‐3p in the DAPK1 sequence. miR‐124‐3p expression was upregulated, and by reducing the protein levels of DAPK1 and GSDMD‐N, pyroptosis was inhibited, indicating that miR‐124‐3p negatively regulates the expression of DAPK1 and exerts an antipyroptotic effect. RES increased the level of miR‐124‐3p and downregulated DAPK1 expression in the pyroptosis model. The levels of the proteins NLRP3, Cleaved caspase‐1 and GSDMD‐N indicated that RES negatively regulated DAPK1 expression by increasing the level of miR‐124‐3p, which affected the NLRP3/Caspase‐11/GSDMD pathway and inhibited microglial pyroptosis. RES decreased GSDMD‐N protein levels in a rat SCI model, and the BBB scores, footprint experiments, HE staining and Nissl staining results indicated that RES promoted injured spinal cord repair. In other words, RES upregulates miR‐124‐3p expression and targets DAPK1 to regulate the NLRP3/Caspase‐11/GSDMD pathway, inhibiting pyroptosis and promoting injured spinal cord repair. This study successfully filled the research gap related to RES and pyroptosis mechanisms in SCI. These achievements may help guide future research on and the development of treatment strategies for SCI. However, a limitation of this study is that although valuable data were obtained through cell‐based experiments and in vivo animal experiments, clinical evidence is lacking, which to some extent affects the validity of the conclusions. Therefore, our team plans to conduct more in‐depth clinical trials in future work to validate and improve upon the results obtained in this study.

## 5 Conclusion

Knocking down DAPK1 affects the NLRP3/Caspase‐11/GSDMD pathway to inhibit pyroptosis in BV2 cells but does not affect the transcription of NLRP3; rather, it alters the activation of the NLRP3 inflammasome. miR‐124‐3p overexpression inhibits BV2 cell pyroptosis by targeting DAPK1. RES upregulates miR‐124‐3p, which targets DAPK1 to regulate the NLRP3/Caspase‐1/GSDMD pathway, thereby inhibiting pyroptosis in BV2 cells and improving functional recovery in rats with SCI. RES inhibits pyroptosis and improves functional recovery in rats with SCI.

## Author Contributions

**Daohui Li:** data curation (equal), methodology (equal), software (equal), validation (equal), writing – original draft (equal), writing – review and editing (equal). **Yongwen Dai:** data curation (equal), formal analysis (equal), methodology (equal), project administration (equal), validation (equal), writing – original draft (equal). **Zhengtao Li:** conceptualization (equal), methodology (equal), validation (equal). **Hangchuan Bi:** data curation (equal), methodology (equal), validation (equal). **Haotian Li:** data curation (equal), methodology (equal), validation (equal). **Yongquan Wang:** formal analysis (equal), software (equal), validation (equal). **Yuan Liu:** software (equal), validation (equal). **Xinpeng Tian:** software (equal), validation (equal). **Lingqiang Chen:** conceptualization (equal), funding acquisition (equal), project administration (equal).

## Conflicts of Interest

The authors declare no conflicts of interest.

## Supporting information


**Figure S1** Mechanism Schematic Diagram.

## Data Availability

The data that support the findings of this study are available from the corresponding author upon reasonable request.
