# Supplementary figures and images for: Resveratrol Upregulates miR‐124‐3p Expression to Target DAPK1, Regulating the NLRP3/Caspase‐1/GSDMD Pathway to Inhibit Pyroptosis and Alleviate Spinal Cord Injury
Source: J Cell Mol Med. 2025 Jan 20;29(2):e70338. doi: 10.1111/jcmm.70338 (PMC11745821; doi:10.1111/jcmm.70338)

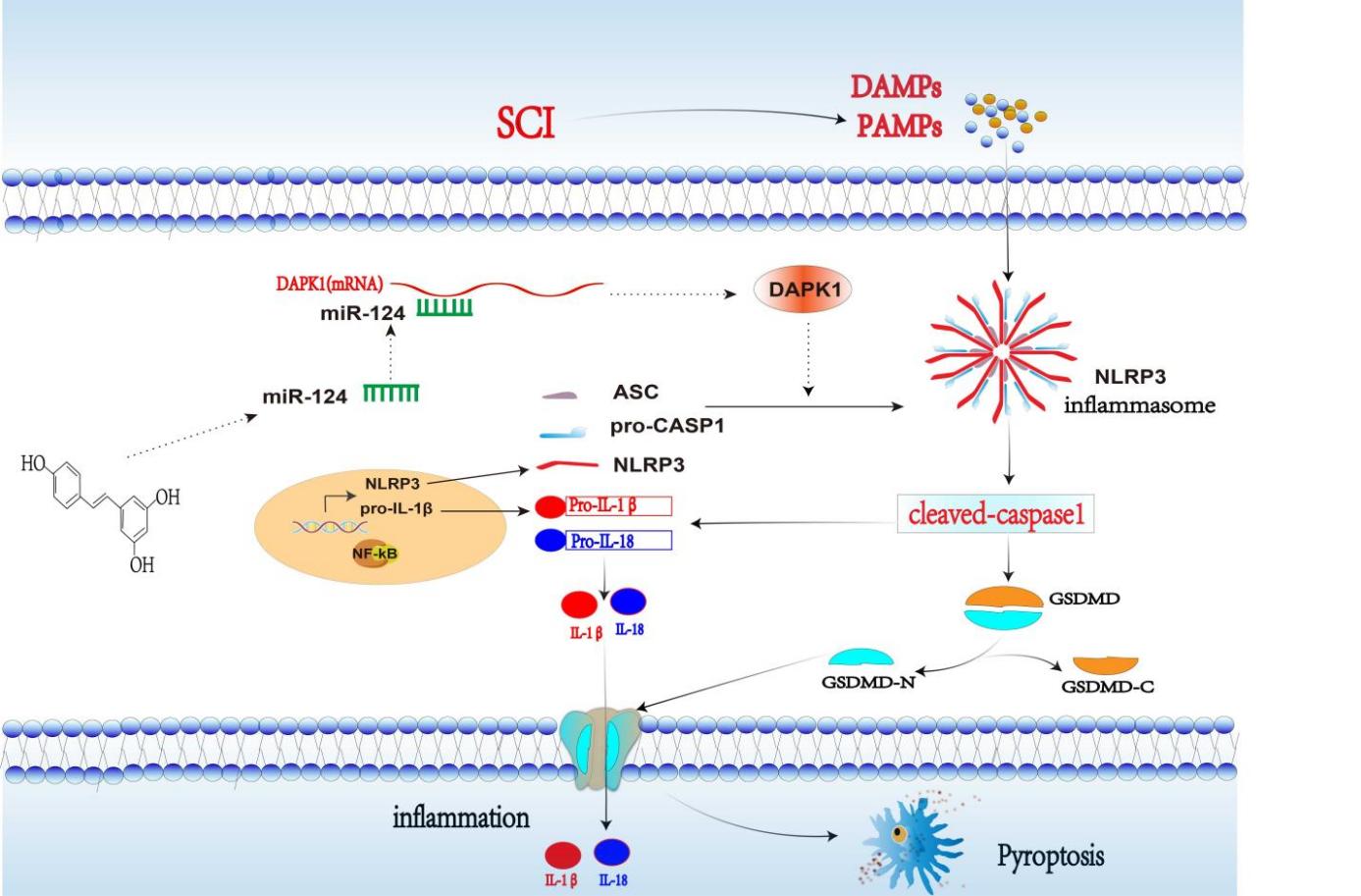

Supplement: Supplementary file 1 — Figure S1 Mechanism Schematic Diagram. [file JCMM-29-e70338-s001.docx]
